# Permeability Coefficient of Concrete under Complex Stress States

**DOI:** 10.3390/ma16124368

**Published:** 2023-06-13

**Authors:** Jiafeng Gu, Qingwen Ren, Mei Tao, Yan Han, Linfei Zhang

**Affiliations:** 1 College of Water Conservancy and Hydropower Engineering, Hohai University, Nanjing 210098, China; hhugjf@126.com (J.G.); lf_zhang@yeah.net (L.Z.); 2 Department of Engineering Mechanics, Hohai University, Nanjing 211100, China; hanyan_hhu@163.com; 3 College of Civil and Architecture Engineering, Chuzhou University, Chuzhou 239000, China; tmei@chzu.edu.cn

**Keywords:** hydraulic structures, concrete, cracking damage, safety assessment, seepage–stress coupling, permeability coefficient, multi-axial loading, volume strain, numerical simulation

## Abstract

Hydraulic structures are typically subjected to long-term hydraulic loading, and concrete—the main material of structures—may suffer from cracking damage and seepage failure, which can threaten the safety of hydraulic structures. In order to assess the safety of hydraulic concrete structures and realize the accurate analysis of the whole failure process of hydraulic concrete structures under the coupling effect of seepage and stress, it is vital to comprehend the variation law of concrete permeability coefficients under complex stress states. In this paper, several concrete samples were prepared, designed for loading conditions of confining pressures and seepage pressures in the first stage, and axial pressures in the later stage, to carry out the permeability experiment of concrete materials under multi-axial loading, followed by the relationships between the permeability coefficients and axial strain, and the confining and seepage pressures were revealed accordingly. In addition, during the application of axial pressure, the whole process of seepage–stress coupling was divided into four stages, describing the permeability variation law of each stage and analyzing the causes of its formation. The exponential relationship between the permeability coefficient and volume strain was established, which can serve as a scientific basis for the determination of permeability coefficients in the analysis of the whole failure process of concrete seepage–stress coupling. Finally, this relationship formula was applied to numerical simulation to verify the applicability of the above experimental results in the numerical simulation analysis of concrete seepage–stress coupling.

## 1. Introduction

Hydraulic concrete structures, such as high concrete dams and concrete linings of high-pressure water transmission tunnels, are at risk of cracking damage and hydraulic splitting under high hydraulic pressure [1,2,3,4,5,6], seriously threatening the safety of the structures. This is due to the fact that the concrete is a kind of porous material with initial internal pores and cracks, which will form seepage channels under hydraulic pressure. Furthermore, the coupling effect of stress and seepage causes changes in the stress state, seepage channels and permeability coefficient of concrete. In addition, numerous researchers [7,8,9,10,11,12,13] have established a correlation between damage and permeability in concrete, demonstrating that the magnitude of damage corresponding to different stress states had a significant influence on the permeability characteristics of concrete.

Nevertheless, in the present safety analysis of hydraulic structures, concrete is usually viewed as an impermeable material [14,15,16], or as a material with a constant permeability coefficient [17,18,19], even though in the seepage–stress coupling analysis, the permeability coefficient varies mainly within the range of the material’s elastic state. Rivera [20] established the permeability coefficient as a function of porosity by groundwater experiments and applied it to geotechnical and concrete materials; Louis [21] established the relationship between the anisotropic permeability coefficient and stress using experimental data from borehole pumping experiments at a dam site. However, they did not consider the change in the permeability coefficient when the concrete was in a damaged condition.

To obtain a thorough and in-depth understanding of the safety assessment of the concrete materials in hydraulic structures, the whole process must be analyzed, beginning with its linear-elastic state and concluding with its failure state. Consequently, it is essential to comprehend the evolution law of the concrete permeability coefficient under complex stress states. Some studies have [22,23] also established relationships between the permeability coefficient and damage variable for rock and concrete, while the damage variables were determined based on stress or strain components. However, their stress states were relatively simple, making it difficult to determine the damage values and permeability coefficients under complex stress states.

This paper employs triaxial seepage–stress coupling experiments to the variation law of the permeability coefficient with volume strain throughout the failure process. The relevant experimental results are applied to the numerical simulation of seepage–stress coupling, providing a reliable basis for parameter selection in the flow–solid coupling analysis of concrete materials.

## 2. Permeability Experiment of Concrete

This chapter covers the triaxial damage experiments for concrete under seepage pressure, which serve as the basis for the subsequent analysis of the interrelationships between the relevant parameters in the following chapters.

### 2.1. Sample Preparation and Experimental Scheme

**Figure 1:**
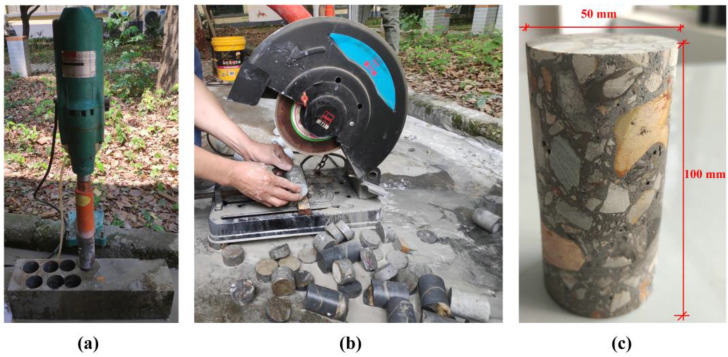
The process of sample preparation. (**a**) Drilling; (**b**) cutting; (**c**) molding.

The samples used in this experiment were made of C30 concrete with the mixture ratio of 0.38:1:1.11:2.72 for water: cement: sand: stone, and the mixture ratio was determined following the specification JGJ 55-2011 [24]. After 28 days of curing, the samples were processed by drilling cores and cutting, resulting in the final molding dimensions of 50 mm in bottom diameter and 100 mm in height, as shown in Figure 1.

It is noteworthy that prior to conducting the experiment, it was necessary to polish the surfaces of the samples using sandpaper to eliminate any apparent roughness and ensure a tight fit with the surrounding sealing rubber sleeve (sleeved on the outside of the samples during the experiment). This enabled complete contact between the upper and lower surfaces of the samples and the rigid loading body within the experimental equipment.

**Figure 2:**
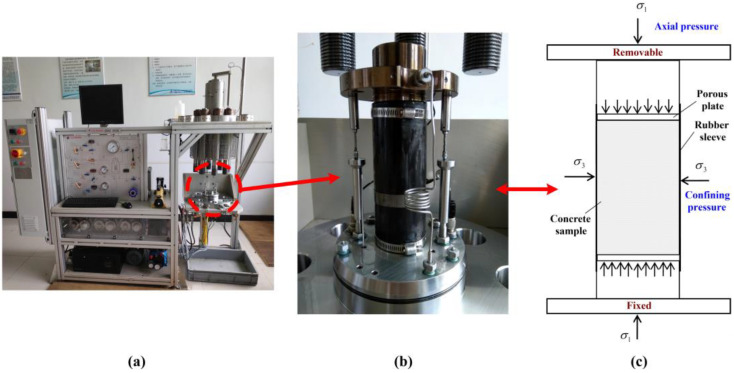
The experimental equipment and local details. (**a**) The experimental equipment; (**b**) the hydraulic chamber in the experimental equipment; (**c**) the force diagram of the hydraulic chamber.

The experimental equipment includes the Triaxial Rock 600-50 Multi-function Mechanical Testing System manufactured by the Top-Industrie company in Vaux-le-Pénil, France. This experimental equipment is capable of simultaneously applying confining pressure, seepage pressure and axial pressure on the sample. Meanwhile, it can also automatically measure and record the amount of seepage under these states. Specifically, during the experiment, the equipment allows hydraulic oil to be pumped from the reservoir into the hydraulic chamber, filling it with oil. Once the chamber is filled, hydraulic oil continues to be pumped until the desired confining pressure is reached. Subsequently, the pump valve is closed to stabilize the confining pressure at the set value. Similarly, water is pumped from the water tank into the corresponding hydraulic chamber, and once the desired seepage pressure is achieved, the corresponding pump valve is closed to maintain a constant value. Additionally, the axial load in this experiment is controlled by constant displacement loading at a specified velocity. See Figure 2 for further details.

The experimental procedure is as follows:

1. Six concrete samples of good quality were selected and numbered as 1^#^ to 6^#^;
2. Before the experiment, the concrete samples were immersed in water to achieve thorough water saturation. The weight before and after immersion was measured, and the porosity of the concrete was calculated based on the weight change. The aggregate content of the concrete samples was calculated based on the density of the aggregate and the density of the mortar material;
3. The samples were placed in the experimental machine, and radial confining pressure was first applied to the samples. After the confining pressure reached the set value and stabilized, the bottom surface was subjected to seepage pressure (water entered from the bottom and exited from the top). The seepage pressure was increased to the set value and stabilized, and the permeability of the concrete under these confining and seepage pressure conditions was calculated. Subsequently, an analysis of the differences was conducted;
4. Axial pressure was gradually applied to the samples until failure, and the axial strain and the amount of seepage during the loading process were measured.

Different confining pressures (10 MPa and 20 MPa) and seepage pressures (1 MPa, 2 MPa and 3 MPa) were applied to samples 1^#^ to 6^#^, and the relevant parameters and applied confining and seepage pressures are shown in Table 1. Furthermore, the specific calculation process regarding the aggregate content of concrete sample is also provided in the note of Table 1.

### 2.2. Measured Value of Permeability Coefficient

In order to quantitatively analyze the permeability of the concrete samples, the following assumptions were made according to the actual situation of the experiment: the seepage in concrete samples was a continuous steady-state flow under constant pressure; the permeating fluid was an incompressible fluid; the concrete sample was a porous medium, with initial pores and micro-cracks uniformly distributed throughout its interior; and during the experiment, the fluid permeation velocity was relatively small, complying with Darcy’s law.

In this experiment, a stable permeation gradient was formed at both ends of the concrete sample. By recording the outflow volume of the permeating fluid every 5 s and according to Darcy’s law, the concrete permeability coefficient K was calculated using Equation (1).

(1) $$K=\frac{LQ\rho g}{A\Delta Pt}$$

where Q is the measured amount of seepage within time t, L is the height of the concrete sample, A is the cross-sectional area of the concrete sample, ΔP is the seepage pressure difference between the two ends of the sample, ρ is the density of the fluid, and g is the gravitational acceleration. In this experiment, L=0.1 m, $A=1.9625\times 10^{-3}{\;m}^{2}$, t=5 s, ΔP=1, 2, 3 MPa, $\rho =1000{\;\mathrm{kg}/m}^{3}$, g=9.8 N/kg, and Q is derived from the experimental equipment data.

## 3. Analysis of Experimental Results

**Figure 3:**
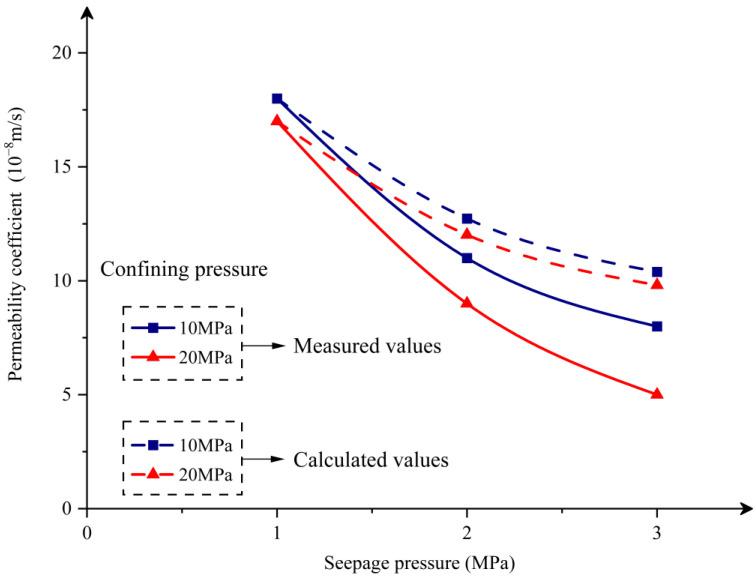
The relationship curves between stable permeability coefficients and confining pressures (10 MPa and 20 MPa) and seepage pressures (1 MPa, 2 MPa and 3 MPa).

Under the combined effect of constant confining pressure and seepage pressure, the amount of seepage of the sample gradually stabilized. The solid lines in Figure 3 show the relationship between the measured permeability coefficient after stabilization and the confining pressure and seepage pressure.

According to the solid lines in Figure 3, the following observations can be made:

1. Under the same seepage pressure and different confining pressures, the permeability coefficient of the sample decreases with increasing confining pressure (the red solid line is below the blue solid line). The magnitude of the reduction increases with increasing seepage pressure, with corresponding reductions of 5.6%, 18.2%, and 37.5% for seepage pressures of 1 MPa, 2 MPa, and 3 MPa, respectively. This is due to the fact that the internal pores and cracks of the concrete sample are gradually compacted and closed under the action of the confining pressure, the seepage channels are narrowed, and the deformation generated within the concrete material acts as a form of obstruction and inhibition to the passage of fluid.
2. Under the same confining pressure and different seepage pressures, the permeability coefficient of the sample decreases with increasing seepage pressure (the red and blue lines in Figure 3 both show a decreasing trend). The reason for this phenomenon can be analyzed from the perspective that the seepage channels in the concrete sample are composed of multiple simple pipes with length l and diameter d (“simple pipes” refer to pipes with constant diameters and no branching). This experiment conforms to the free outflow characteristic in the hydraulic calculation of simple pipes, and the expression for the flow rate Q through a single simple pipe is Equation (2) [25].
  (2) $$Q=\mu_{c}A^{\prime }\sqrt{2gH}$$
  where $A^{\prime }$ is the cross-sectional area of the pipe for water flow, H is the difference in water heads, and $\mu_{c}$ is the flow coefficient of the pipeline system, determined by the following equation:
  (3) $$\mu_{c}=\frac{1}{\sqrt{1+\lambda \frac{l}{d}+\sum \zeta }}$$
  where λ is the coefficient of friction along the pipe, which is a function of the fluid Reynolds coefficient Re, and ∑ζ is the sum of the local head loss coefficients in the pipe. We can assume that the flow rates of the concrete sample in different head differences $H_{1}$ and $H_{2}$ (i.e., different seepage pressures $\Delta P_{1}$ and $\Delta P_{2}$) are $Q_{1}$ and $Q_{2}$, respectively. For the same concrete sample under different head differences, it can be assumed that λ and ∑ζ do not change, and thus the flow coefficient of the pipeline system $\mu_{c}$ does not change, which results in Equation (4).
  (4) $$\frac{Q_{1}}{Q_{2}}=\sqrt{\frac{H_{1}}{H_{2}}}$$
  where $\frac{H_{1}}{H_{2}}=\frac{\Delta P_{1}}{\Delta P_{2}}$ (ΔP is the seepage pressure), which in turn leads to Equation (5).
  (5) $$\frac{Q_{1}}{Q_{2}}=\sqrt{\frac{\Delta P_{1}}{\Delta P_{2}}}$$
  According to Equation (1) obtained from Darcy’s law, Equation (5) can be written as follows:
  (6) $$\frac{K_{1}}{K_{2}}=\sqrt{\frac{\Delta P_{2}}{\Delta P_{1}}}$$
  It can be observed that the permeability coefficient of the concrete sample is inversely proportional to the square root of the seepage pressure, i.e., the greater the seepage pressure, the smaller the permeability coefficient. The dashed lines in Figure 3 represent the calculated values obtained using Equation (6) (assuming that the calculated value when the seepage pressure is 1 MPa is the same as the measured value). The reason for the discrepancy between the calculated values (dashed lines) and measured values (solid lines) is that some assumptions are made in the derivation of Equation (6). However, the trend of decreasing permeability coefficient with increasing seepage pressure is the same for both calculated and measured values.

**Figure 4:**
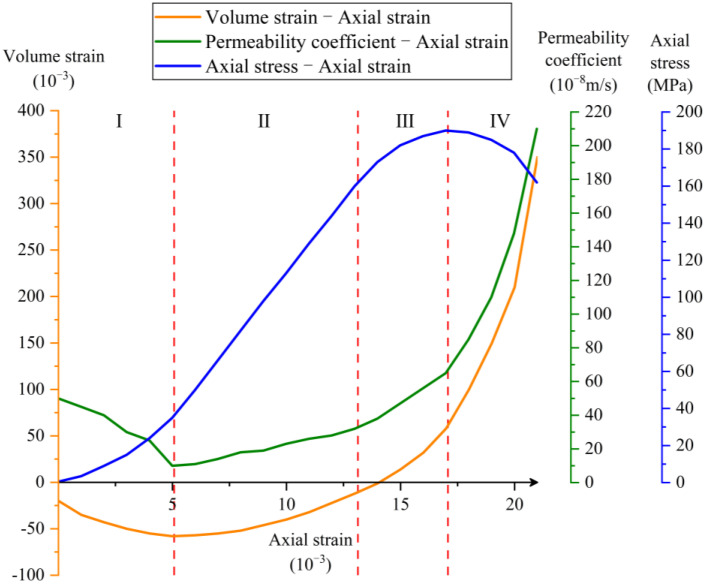
Volume strain/permeability coefficient/axial stress–axial strain curves.

The sample was subjected to a certain confining pressure and seepage pressure, and axial pressure was applied after the permeability coefficient was stabilized. As the axial pressure increased, the micro-structure in the sample gradually changed, and the permeability coefficient correspondingly changed. Figure 4 presents the relationship curve between the permeability coefficient and axial strain of sample 1^#^ (green line), and simultaneously plots the stress–strain curve of the loading process (blue line).

The variation in the permeability coefficient of the concrete sample can be divided into four stages (Stage I to Stage IV, divided by vertical red dashed lines in Figure 4) as follows:

I. After applying axial compression, the compressive stress starts increasing from zero. With the increase in deformation, the stress–strain curve exhibits an ascending trend with a progressively accelerating rate, revealing the material’s inherent “locking” effect. This effect is attributed to the gradual closure of the pores and micro-cracks existing in the concrete. Therefore, the permeability coefficient decreases with the increase in axial strain.
II. The stress–strain curve is basically linearly elastic. Although the permeability coefficient increases with the increase in axial strain, the change is not significant, and it remains relatively stable.
III. The stress–strain curve enters the strengthening stage. As the axial strain increases, the permeability coefficient increases significantly at a higher rate. This is because the concrete begins to become damaged in this stage, and internal micro-cracks and pores increase. Meanwhile, new cracks emerge and connect, resulting in an increase in seepage channels.
IV. This stage is the softening stage of the stress–strain curve. As the axial strain continues to increase, macroscopic cracks appear in the concrete, leading to a sharp increase in the permeability coefficient. The sudden increase in the permeability coefficient occurs roughly near the peak point of the stress–strain curve. In the later part of this stage, the change in the permeability coefficient becomes extremely unstable and discontinuous; thus, the permeability coefficient calculated at this time no longer conforms to the assumption of Darcy’s law and is not plotted in Figure 4.

## 4. Relationship between Permeability Coefficient and Volume Strain

### 4.1. Relationship Based on Experimental Results

The relationship between the permeability coefficient and uniaxial strain is difficult to apply in seepage–stress coupling analysis under complex stress states. Existing research results [22,23] have shown that for porous medium materials, such as rock mass, the permeability characteristic was closely related to the volume changes inside the material. In this section, the relationship between the permeability coefficient and volume strain is investigated based on experimental results.

Volume strain is an invariant that does not change with the coordinate system and can reflect complex stress states. The volume strain $\varepsilon_{V}$ is given by the following equation:

(7) $$\varepsilon_{V}=\varepsilon_{r}+\varepsilon_{\theta }+\varepsilon_{z}$$

where $\varepsilon_{r}$, $\varepsilon_{\theta }$, and $\varepsilon_{z}$ represent the radial, circumferential, and axial strains, respectively. The cylindrical sample used in this experiment is axisymmetric. Therefore, in the three-dimensional coordinate system, the force acting on the sample under the combined effect of confining pressure, axial pressure, and seepage pressure is also axisymmetric. In this case, $\varepsilon_{r}=\varepsilon_{\theta }$, and thus the above equation, can be written as follows:

(8) $$\varepsilon_{V}=2\varepsilon_{\theta }+\varepsilon_{z}$$

where $\varepsilon_{\theta }$ and $\varepsilon_{z}$ can be measured and calculated by the experimental machine. Positive volume strain indicates volume expansion, while negative value indicates volume compression. The relationship curve between volume strain and axial strain is also plotted in Figure 4 (orange line).

The four stages (I, II, III, and IV) shown in Figure 4 correspond to the volume strain of “negative, negative, transitioning from negative to positive, and positive”, indicating that the experimental sample experienced the following four processes: compression after applying axial stress, gradual reduction in the compression rate, gradual expansion, and overall expansion. 

During Stage I, the volume strain of the concrete sample gradually decreases and reaches a minimum value, indicating that the compression amount gradually increases and reaches the compression limit. At the minimum volume strain, the permeability coefficient reaches its minimum value. During Stage II, the volume strain of the concrete sample gradually increases, and the value of permeability coefficient increases slowly. During Stage III, the volume strain changes from negative to positive, and the value of permeability coefficient gradually increases at an accelerated rate. In the first part of Stage IV, the volume strain of the concrete sample increases rapidly, and the value of permeability coefficient also increases rapidly. See Table 2 for details.

It can be observed that there is a significant correlation between the concrete permeability coefficient and volume strain, with the two variables exhibiting a strong positive relationship. During the stage of volume compression, the volume strain becomes negative, with a smaller volume strain indicating greater compression, leading to a smaller value of the permeability coefficient. Conversely, during the stage of volume expansion, the volume strain becomes positive, with a larger volume strain indicating greater expansion, resulting in a greater value of the permeability coefficient.

**Figure 5:**
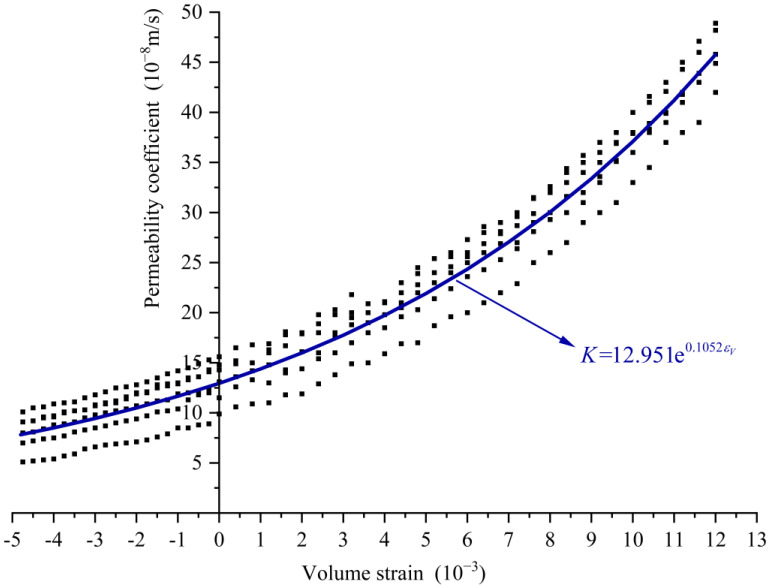
Permeability coefficient–volume strain scatter plot and fitted curve.

The measured points of volume strain and permeability coefficient for samples 1^#^ to 6^#^ are plotted in Figure 5, and the following curve in the form of exponential function is used to fit the experimental data points:

(9) $$K=K_{0}e^{\alpha \varepsilon_{V}}$$

where $K_{0}$ represents the initial value of the permeability coefficient, which is the value of K when $\varepsilon_{V}=0$; α is a constant parameter. $K_{0}$ and α can be obtained by fitting. By fitting all the measured points in Figure 5, the value of $K_{0}$ is obtained as 12.951, the value of α is 0.1052, with a goodness of fit value of $R^{2}=0.99$ and the above equation can be written as follows:

(10) $$K=12.951e^{0.1052\varepsilon_{V}}$$

Equation (10) provides the relationship between the concrete permeability coefficient and volume strain. Based on this equation, the permeability coefficient corresponding to any moment during the whole loading process of the concrete can be determined by the volume strain at that moment.

**Figure 6:**
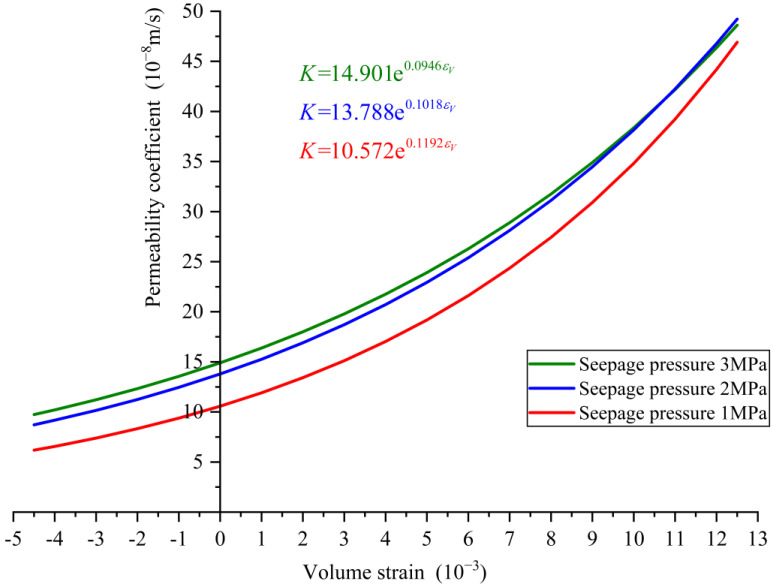
The fitted relationship curves between permeability coefficient and volume strain (seepage pressure is 1 MPa, 2 MPa and 3 MPa, respectively).

If considering the influence of different seepage pressures on concrete samples, the relationships between the permeability coefficient and volume strain under seepage pressures of 1 MPa, 2 MPa, and 3 MPa can be obtained separately, denoted as the following Equations (11)–(13), as shown in Figure 6.

(11) $$K=10.572e^{0.1192\varepsilon_{V}}$$

(12) $$K=13.788e^{0.1018\varepsilon_{V}}$$

(13) $$K=14.901e^{0.0946\varepsilon_{V}}$$

The goodness of fit is $R^{2}=0.9925$, $R^{2}=0.9897$, and $R^{2}=0.9705$, respectively. Therefore, the permeability coefficient corresponding to the volume strain at any time during the whole loading process of concrete under different seepage pressures can be obtained based on the above relationships.

### 4.2. Application of Dynamic Variation in Permeability Coefficient in Numerical Simulation

In most numerical simulations of concrete seepage–stress coupling, the concrete permeability coefficient is usually taken as a constant value, and this fixed seepage field is applied to the calculation model [26,27,28,29,30]. This approach cannot effectively consider the influence of stress field changes on the variation in seepage; therefore, it is necessary to consider the dynamic change process of the permeability coefficient during the calculation. In this section, the relationship between the permeability coefficient and volume strain obtained previously is applied to the numerical simulation of seepage–stress coupling, and compared and analyzed with the previously obtained experimental results.

The numerical model used in this section is identical to the concrete sample experimented, which is cylindrical in shape with a bottom diameter of 50 mm and a height of 100 mm. The elastic modulus of concrete E=30,000 MPa, the density $\rho =2375{\;\mathrm{kg}/m}^{3}$ (measured experimentally), the initial porosity $n_{0}=0.047$ (the average value of $p_{orig}$ in Table 1), the compressive strength $f_{c}=20.1\;\mathrm{MPa}$, tensile strength $f_{t}=2.01\;\mathrm{MPa}$, and Poisson’s ratio v=0.167. The biaxial compressive strength $f_{bc}$ is taken as 1.2 times the uniaxial compressive strength, i.e., $f_{bc}=24.1\;\mathrm{MPa}$.

The fluid continuity differential equation can be written as follows:

(14) $$\nabla \cdot \left(\rho v\right)+\frac{\partial \left(\rho \phi \right)}{\partial t}=\rho q$$

where ∇ is the Hamiltonian operator, v is the vector of permeation velocity, ϕ is the porosity, and q is the intensity if there is a “source” distribution within the control volume. The detailed derivation process and interpretation can be found in reference [31].

If Darcy’s law is formally extended to three-dimensional fluids for an isotropic medium, the equation can be written as follows:

(15) $$v=-\frac{k}{\mu }(\nabla p-\rho g)$$

where μ is the coefficient of dynamic viscosity (unit: Pa·s) and represents the inherent property of a liquid. For water at a temperature of 20 °C, $\mu =1\times 10^{-3}\;\mathrm{Pa}\cdot s$; p is the porewater pressure (equivalent to the seepage pressure in this paper); k is the penetration rate (unit: m^2^), and the conversion between k and permeability coefficient K can be achieved using the following equation:

(16) $$K=\frac{k\rho g}{\mu }$$

The stress–strain constitutive equation for concrete under the influence of porewater pressure can be written as follows:

(17) $$\sigma_{ij}^{\prime }=D_{ijkl}\varepsilon_{kl}-\alpha p\delta_{ij}$$

where $\sigma_{ij}^{\prime }$ is the tensor of effective stress, $D_{ijkl}$ is the tensor of the elastic modulus, $\varepsilon_{kl}$ is the tensor of strain, α is the Biot coefficient; $\delta_{ij}$ is the Kronecher symbol (when i=j, $\delta_{ij}=1$ and when i≠j, $\delta_{ij}=0$).

**Figure 7:**
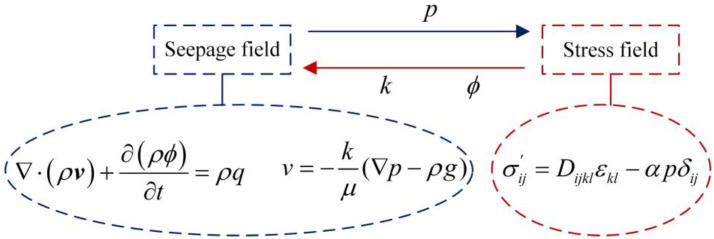
Schematic diagram of seepage–stress coupling mechanism.

Based on the fluid continuity equation, Darcy’s law and the stress–strain control equation mentioned above, the schematic diagram of the seepage–stress coupling mechanism as shown in Figure 7 can be obtained.

As can be observed from Figure 7, the influence of the seepage field on the stress field is mainly reflected in the change in porewater pressure p caused by the variation in the seepage field, which in turn changes the stress field inside the concrete. On the other hand, the influence of the stress field on the seepage field is manifested as structural deformation, leading to changes in the porosity ϕ and the penetration rate k, thereby altering the seepage field inside the concrete.

**Figure 8:**
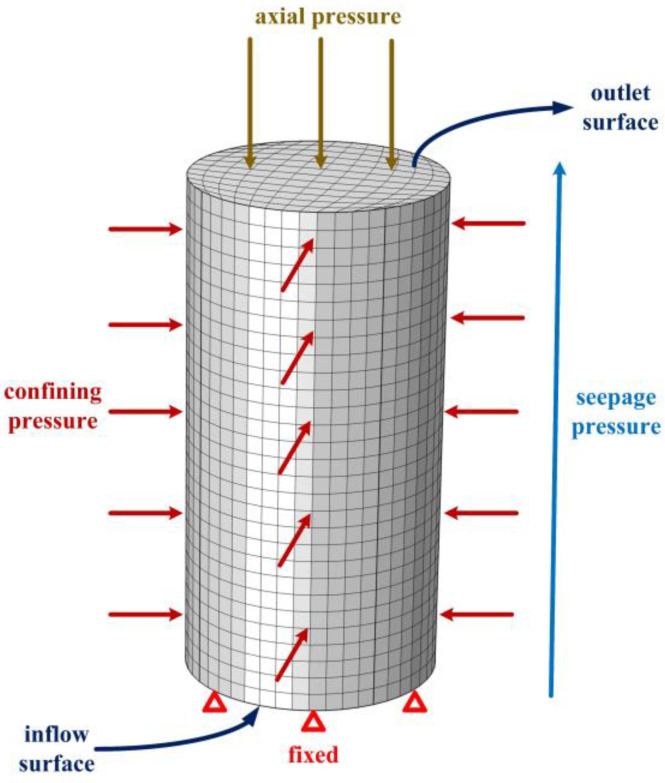
Numerical calculation model.

The calculation model is shown in Figure 8. Confining pressure is applied around the model, while seepage pressure is applied at the top and bottom ends with water inflow at the bottom and outflow at the top. The model is impermeable on all other surfaces. The bottom end is fixed, while a downward displacement load (i.e., compression load) is applied at the top end. The calculation conditions are the same as those presented in Table 1, with compressive displacement used as the axial load. To ensure convergence of the calculation, the final compression displacement is controlled to be within the range of 0.08 to 0.09 mm. The simulation is carried out using COMSOL Multiphysics, considering the variations in porosity ϕ (as given by Equation (18)) [32] and permeability coefficient K (as described in Section 4.1).

(18) $$\phi =1-\frac{1-n_{0}}{1+\varepsilon_{v}}$$

**Figure 9:**
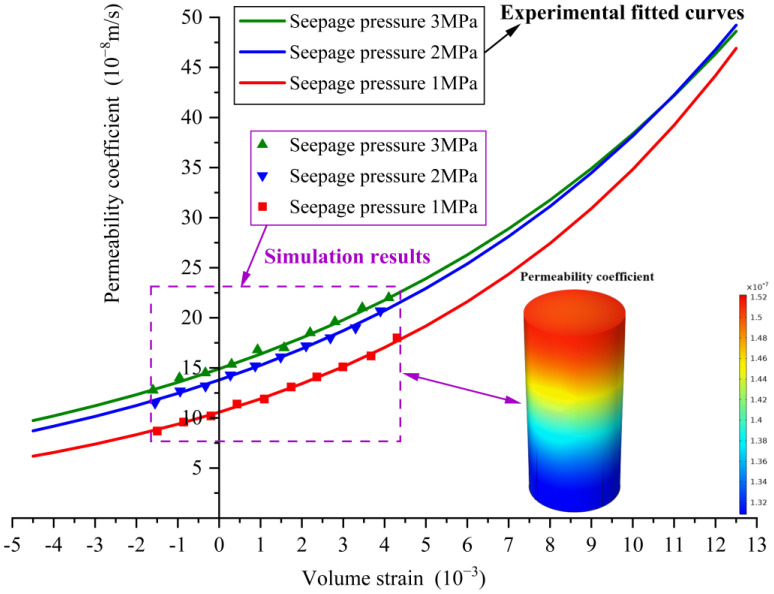
Permeability coefficient–volume strain curves (experimental results), scatter plot (simulation results) and permeability coefficient nephogram.

The displacement load is divided into 100 steps, and the overall permeability coefficient and volume strain of the calculation model after each step of the displacement load application are exported and plotted in Figure 9. In Figure 9, the overall permeability coefficient nephogram of the concrete calculation model at 80th load step is also plotted. Figure 9 indicates that the numerical simulation data points for the relationship between the permeability coefficient and volume strain are in close proximity to the fitted curves obtained from the experimental data in Section 4.1. This demonstrates that the relationship curves obtained from the experiments can be effectively applied in the numerical simulation of concrete seepage–stress coupling analysis.

## 5. Conclusions

This paper mainly studies the variation law of the concrete permeability coefficient under complex stress states. Different loading conditions with various confining pressures and seepage pressures were designed to carry out permeability experiments on concrete material under multi-axial loading by applying axial compression. The results demonstrate a close correlation between the permeability characteristics of concrete and its stress states (axial pressure, confining pressure, and seepage pressure). The relationship between the permeability coefficient and volume strain is derived, enabling the determination of the permeability coefficient under complex stress states. The above results can be utilized for seepage–stress coupling analysis of hydraulic concrete structures.

The following are the key conclusions obtained from the full paper:

1. Qualitative and quantitative variations in the concrete permeability coefficient under different confining pressures and seepage pressures are revealed, and are explained in terms of both the mechanism and formula derivation.
2. The variation law of the concrete permeability coefficient under axial compression is revealed, and is described in stages.
3. A quantitative expression of the relationship between the concrete permeability coefficient and volume strain is established, which can provide a scientific basis for the determination of the permeability coefficient in the analysis of the whole failure process of concrete seepage–stress coupling.
4. The result of Conclusion 3 is applied to the numerical simulation of concrete seepage–stress coupling, and the result shows that it has good applicability in the subsequent numerical simulation analysis.

Meanwhile, as a representative porous medium material, the research method for the permeability characteristics of concrete presented in this paper can also serve as a reference for the study of gas or liquid permeability characteristics of other porous materials.

## Figures and Tables

**Table 1:** Parameters and experimental conditions of concrete samples.

Sample	$m_{0}$	$m_{1}$	$p_{orig}$	$\alpha_{agg}$	Seepage Pressure (MPa)	Confining Pressure (MPa)
1^#^	457.3	466.3	0.046	0.460	1	10
2^#^	458.7	467.9	0.047	0.479	2	10
3^#^	458.6	467.7	0.046	0.478	3	10
4^#^	459.0	468.3	0.047	0.483	1	20
5^#^	457.6	466.8	0.047	0.464	2	20
6^#^	453.3	462.5	0.047	0.405	3	20

Note: (1) $m_{0}$ is the initial quantity, $m_{1}$ is the quantity with water saturation, $p_{orig}$ is the original porosity, $\alpha_{agg}$ is the aggregate content of the concrete sample; (2) $\alpha_{agg}$ is calculated as follows: evidently, $m_{0}=m_{agg}+m_{mor}=\rho_{agg}V\alpha_{agg}+\rho_{mor}V(1-\alpha_{agg})$, $m_{agg}$ is the quantity of the aggregate in the sample, $m_{mor}$ is the quantity of the mortar material in the sample, $\rho_{agg}$ is the density of the aggregate with an experimental measurement value of $2530{\;\mathrm{kg}/m}^{3}$, $\rho_{mor}$ is the density of the mortar material with an experimental measurement value of $2160{\;\mathrm{kg}/m}^{3}$, V is the volume of the sample; subsequently, $\alpha_{agg}$ can be obtained from the aforementioned equation.

**Table 2:** Correspondence table between permeability coefficient and volume strain change for each stage.

Stage	Sign of Volume Strain	Change in Volume	Change in Permeability Coefficient
I	Negative	Compressed continuously	Decreased, and to minimum
II	Negative	Starts to expand, but overall compressed	Starts to increase slowly
III	Negative to positive	Expanded continuously	Accelerated increase
IV	Positive	Overall expanded	Increase rapidly

## Data Availability

Not applicable.
